# Predicting the effects of eutrophication mitigation on predatory fish biomass and the value of recreational fisheries

**DOI:** 10.1007/s13280-019-01263-1

**Published:** 2019-10-09

**Authors:** Göran Sundblad, Lena Bergström, Tore Söderqvist, Ulf Bergström

**Affiliations:** 1 Department of Aquatic Resources, Institute of Freshwater Research, Swedish University of Agricultural Sciences (SLU), Stångholmsvägen 2, 178 93 Drottningholm, Sweden; 2 Department of Aquatic Resources, Institute of Coastal Research, Swedish University of Agricultural Sciences (SLU), Skolgatan 6, 742 42 Öregrund, Sweden; 3 Anthesis Enveco, Barnhusgatan 4, 111 23 Stockholm, Sweden

**Keywords:** Economic value, Ecosystem services, Eutrophication, Fisheries, Species distribution model, Travel cost method

## Abstract

Improving water clarity is a core objective for eutrophication management in the Baltic Sea, but may influence fisheries via effects on fish habitat suitability. We apply an ensemble of species distribution models coupled with habitat productivity functions and willingness-to-pay estimates to assess these effects for two coastal predatory fish species, European perch (*Perca fluviatilis*) and pikeperch (*Sander lucioperca*). The models predicted a 37% increase in perch and 59% decrease in pikeperch biomass if reaching the reference level for water clarity in the Baltic Sea Action Plan. Reaching the target level was predicted to increase perch biomass by 13%. However, the associated economic gain for the recreational fisheries sector was countervailed by an 18% pikeperch reduction. Still, a net benefit was predicted since there are six times more fishing days for perch than pikeperch. We exemplify how ecological modelling can be combined with economic analyses to map and evaluate management alternatives.

## Introduction

Increasing pressure from human activities requires environmental strategies and policies that can ensure a sustained delivery of ecosystem services (Costanza et al. 1997; Halpern et al. 2015). Supporting science-based evaluations of policy development and implementation, coupled scenario analyses provide potentially useful tools for integrating ecology, social science and policymaking (Coreau et al. 2009). By evaluating the effects of changes in environmental forcing (including human-induced pressures) in spatially explicit, process-based ecological models, the development of ecosystem functions and services can be explored for a set of plausible futures, i.e. scenarios (Qiu et al. 2018). Extending the analysis to include expected net changes in the economic value of benefits provisioned by the ecosystem under different scenarios opens the possibility to explore economic benefits and trade-offs (Stål et al. 2008; Costanza et al. 2014; Bauer et al. 2018).

As a key approach to improve environmental management of the Baltic Sea, the Baltic Marine Environment Protection Commission (HELCOM) has adopted the Baltic Sea Action Plan (BSAP; HELCOM 2007; Backer et al. 2010), to be updated by 2021. The plan is dedicated to turning scientific knowledge into strategic policy implementation, focusing on four themes; eutrophication, biodiversity, hazardous substances and maritime activities.

Among these, eutrophication is a major problem in the Baltic Sea as a consequence of long-lasting inputs of nitrogen and phosphorus since the mid-1900s, which to a large extent remain in the basin due to its semi-enclosed nature (Andersen et al. 2015). A core indicator for following up on the status of eutrophication is water clarity, which represents the water’s permeability to light, measured as the Secchi depth during summer (HELCOM 2018a, b). Water clarity is a suitable indicator of eutrophication since it shows a strong relationship with chlorophyll *a* and the abundance of pelagic primary producers in the water column, which benefit from elevated nutrient levels at sea and are indicative of eutrophication (Fleming-Lehtinen and Laamanen 2012). Threshold values for the water clarity indicator are sub-basin specific, and are set based on scientific evaluation and common agreement among countries around the Baltic Sea (HELCOM 2018a, b). The BSAP sets reference levels for water clarity based on historical data, and target levels to 25% deviation from the reference level (HELCOM 2007; Backer et al. 2010).

Nutrient loading to the Baltic Sea is currently decreasing in response to reduction measures (HELCOM 2018a). Eutrophication at sea is also being relieved in some aspects, even though several challenges remain due to pollution legacies as well as biotic and abiotic processes in the ecosystem (Andersen et al. 2015; HELCOM 2018a). The elevated nutrient conditions increase the production of ephemeral primary producers, such as phytoplankton and filamentous algae, which leads to shading and impaired growth conditions for larger, habitat-forming vegetation, such as bladderwrack and perennial macrophytes (Berger et al. 2004; Austin et al. 2017), and subsequent deterioration of associated ecosystem services (Rönnbäck et al. 2007). For example, many fish species rely on the habitats shaped by structurally complex vegetation during early life stages, and therefore degradation or loss of vegetated habitats is associated with negative effects on population abundances (Mumby et al. 2004; Seitz et al. 2013; Hansen et al. 2018).

However, responses to changes in eutrophication can be species specific. The two percid fishes, Eurasian perch (*Perca fluviatilis*; hereafter perch) and pikeperch (*Sander lucioperca*) are both important species for commercial and recreational fishing in the Baltic Sea (Lehtonen et al. 1996; Ådjers et al. 2006). These large predatory fishes may through their predation, leading to a trophic cascade decreasing the growth of filamentous algae, indirectly relieve the ecosystem symptoms of eutrophication (Östman et al. 2016; Donadi et al. 2017). Both species spawn in spring and juveniles spend their first summer in shallow, sheltered and warm inlets and bays (Lehtonen et al. 1996; Snickars et al. 2010). One important difference between them is their adaptation to water clarity. Perch prefers clear water and pikeperch more turbid environments, such as those created under elevated nutrient conditions (Sandström and Karås 2002; Ljunggren and Sandström 2007; Veneranta et al. 2011), suggesting contrasting population level effects of reducing symptoms of eutrophication (Bergström et al. 2013).

Overall, eutrophication mitigation appears to provide economic net benefits. A recent cost-benefit analysis of a cost-effective international nutrient abatement programme meeting BSAP objectives indicated an annual net gain amounting to about € 2300 million (Scharin et al. 2016). Regarding recreational activities in the Baltic Sea, estimates based on travel cost approaches for its nine bordering states suggest that the total annual recreation benefits are close to € 15 billion, but could increase by 7–18% under a water quality improvement scenario (Czajkowski et al. 2015). Transdisciplinary models also suggest net benefits of nutrient load reductions for the commercial fisheries in Baltic Sea offshore areas, and the use of spatial models highlight geographical differences (Bauer et al. 2018). However, the impact on the recreational fishery and the associated economic value is less known.

Since reducing eutrophication is a slow and in many cases complex process, it is necessary to examine potential consequences of mitigation measures on species, functions and ecosystem services to support effective and relevant measures. Furthermore, as ecosystem responses to eutrophication vary geographically, it is important to take spatial variability into account (Bergström et al. 2013; Bauer et al. 2018). Species distribution models provide a tool which can predict potential effects of changes in eutrophication in higher geographical detail. In these models, the distribution of species is related to a set of explanatory variables describing the biophysical environment of the species, such as depth, wave exposure and Secchi depth. These environmental variables are then used to predict the distribution of a species on a map (Elith and Leathwick 2007). The use of species distribution models in research and management has developed quickly during the last decade and a variety of fundamentally different modelling methods are available, each with their strength and weaknesses (e.g. Bučas et al. 2013). To utilize the strengths of conceptually different modelling techniques, several methods can be combined in an ensemble approach (Araújo and New 2007).

Here, we evaluate quantitative scenarios for changes in water clarity to assess the chain of events from eutrophication mitigation to potential effects on coastal fish distribution and biomass, and the associated economic value for recreational fisheries. We achieve this by applying species distribution models in an ensemble approach, evaluating different scenarios, which we combine with habitat productivity functions (Sundblad et al. 2014) and an economic assessment on willingness-to-pay (Söderqvist et al. 2005). The analyses are concentrated around the BSAP core indicator water clarity, which is a key predictor of suitable recruitment habitats for perch and pikeperch (Bergström et al. 2013). In addition, by economically valuing the changes in biomass from a recreational fisheries perspective, we aim at indicating the economic net impact of this particular effect of combatting eutrophication. In so doing we illustrate how cross-disciplinary approaches can contribute with scientific knowledge in support of management in line with a sustainable development.

## Materials and methods

### Scenarios

The eutrophication scenarios are rooted in the Baltic Sea Action Plan environmental objective “a Baltic Sea unaffected by eutrophication” (HELCOM 2007; Backer et al. 2010), represented by different values for the core indicator on water clarity (HELCOM 2018a). Seven scenarios for effects on species distributions were applied as in Bergström et al. (2013), designed as follows: (1) the situation by the onset of the Baltic Sea Action Plan in 2007 (0% change, hereafter termed initial conditions); (2) a slightly deteriorating condition (10% decrease in Secchi depth) and (3) five scenarios representing improved water clarity (11, 20, 30, 40 and 48% increase in Secchi depth). Levels 11 and 48% correspond to the target and reference levels for the Baltic Proper stated in the original Baltic Sea Action plan (HELCOM 2007; Backer et al. 2010; Bergström et al. 2013). The target levels have later been revised (HELCOM 2018b), but involved only minor changes compared to Backer et al. (2010; Table 1).

### Ecological modelling

The species distribution models identified potential recruitment habitats for perch and pikeperch based on field surveys of spawning and young-of-the-year fish (Bergström et al. 2013). The species-environment relationships, including water clarity as estimated by the Secchi depth, were modelled using three different statistical modelling techniques: GAM, Maxent and random forest (Wood 2006; Cutler et al. 2007; Phillips and Dudík 2008). The predicted recruitment habitats for the two species under the different scenarios for water clarity and using three modelling techniques rendered in total 42 recruitment habitat maps (25 m cell resolution, Bergström et al. 2013). Compared to Bergström et al. (2013), this study was limited to Swedish waters (Counties of Södermanland, Stockholm and Uppsala, see Fig. 2) in order to overlap with the economic data (below).

### Habitat productivity functions

Habitat productivity functions, which describe the relationship between the amount of recruitment habitat and the density of large fish (Sundblad et al. 2014), were combined with the 42 recruitment habitat maps (Bergström et al. 2013), to predict the biomass of large perch (> 20 cm) and pikeperch (> 30 cm) under the different scenarios. This was done in two steps. First, for every cell in each of the 42 maps we estimated habitat availability as a proportion (the area predicted to function as recruitment habitat divided by the total water area) within typical maximum migration distances. Typical migration distances were defined as the distances within which 80% of perch and 75% of pikeperch are recaptured based on tagging studies; 10 km for perch and 15 km for pikeperch (Saulamo and Neuman 2002; Sundblad et al. 2014). Thereby, each cell in the resulting raster expressed the amount of habitat within the migration distance of that cell. The calculations were made using focal statistics in ArcGIS, Esri. Unlike Sundblad et al. (2014), migration barriers, i.e. islands and land, could not be taken into account, since computational constraints restricted the use of cost distance functions from each cell (instead of from each population as applied in Sundblad et al. 2014). As a consequence, the focal search window potentially over- or under-estimated habitat availability, depending on the amount of barriers and the amount of habitat that then could, or could not, be reached. This was apparent at the very local scale, while it should be of less importance for the study area as a whole.

Secondly, based on the estimated habitat availability under different scenarios, habitat productivity functions as presented in Sundblad et al. (2014) were used to calculate the expected catch-per-unit-effort (CPUE, number of fish per net and night) of adult perch and pikeperch (> 20 and > 30 cm, respectively). The habitat productivity functions were CPUE = 2.03 (± 0.69 SE) * ln(*x*) + 9.39 (± 1.27 SE) for perch (*n *= 12, *p *= 0.015, *r*^2^= 0.46), and CPUE = 0.05 (± 0.02 SE) * ln(*x* + 0.02) + 0.21 (± 0.04 SE) for pikeperch (*n *= 12, *p *= 0.013, *r*^2^= 0.48), where *x* was the habitat availability calculated in the first step. The analyses resulted in 42 new maps, in which each cell showed the expected CPUE of large predatory fish hypothetically applying a standardized gill net test fishing in that cell. Lastly, the average and associated uncertainty (1 standard error, SE), of the three modelling techniques was calculated for each eutrophication level scenario.

The predicted CPUE of large perch and pikeperch were evaluated against observed catches in standardized gill nets based on data from 11 existing monitoring sites in the study area (locations of sites are shown in Fig. 2). Three of these sites had also been utilized in the development of the habitat productivity functions (Sundblad et al. 2014), but here included more years (2002–2016) compared to the previous study (2005–2006). For the evaluation of pikeperch, one site was excluded as it was no longer representative to the conditions under which the habitat productivity functions were developed, due to implementation of a total fishing ban in 2010–2015 (Bergström et al. 2016). Values for CPUE were converted to biomasses (kg ha^−1^) using existing conversion functions (Heibo and Karås 2005), and information on the average weight of a perch > 20 cm and a pikeperch > 30 cm, respectively, for the gear type used (information from the national fish monitoring database KUL). Based on data on abundances and weights per length group, the average weight of a perch (> 20 cm) was estimated to 0.23 kg (*n *= 10 941), and 0.51 kg for pikeperch (> 30 cm, *n *= 355). Since coastal perch is primarily found at 0–10 m depths, observed CPUE in monitoring and all maps have been limited to these depths for perch (Fig. 2).

### Economic analyses

The effect on opportunities for recreational fishing of perch and pikeperch was monetized using data from Söderqvist et al. (2005), who estimated the recreational value of fishing in the study area in 2002 through applications of the travel cost method (TCM). The TCM is a revealed preference method that makes use of travel data obtained through surveys for estimating people’s demand for visiting various recreational sites (Freeman et al. 2014). As the expected catches of a fish species varies across different sites, data on travels, associated travel costs and the variation in catches can allow an estimation of people’s economic trade-offs between travel costs and catches, expressed by people’s willingness-to-pay (WTP) for an increased catch. In the study by Söderqvist et al. (2005), randomly selected members of the Swedish Anglers Association (*n *= 2000) and the general public (*n *= 2000) living in the counties of Uppsala and Stockholm replied to mail questionnaires relating to their use of the archipelago, including information about visits to sites in the study area, their fishing at those sites, the distance travelled, travel time, travel costs and catches for different fish species (expressed as weight-per-unit-effort, WPUE). These data allowed the quantification of explanatory variables in conditional logit models predicting the probability that a particular fishing site is selected, and coefficients from the estimated models were subsequently used for computing the WTP for a changed WPUE (see Söderqvist et al. 2005 for details). Finally, the results on individual WTP was related to another survey, conducted annually since 2013 by Statistics Sweden, focusing on the recreational fishing habits among the Swedish general public (Swedish Agency for Marine and Water Management, SwAM 2019). From the national survey we calculated the relative species preference as the ratio of the fishing effort for perch to the fishing effort for pikeperch. Specifically, using yearly averages (2013–2017) and associated uncertainty (95% CI) from the Baltic proper, effort was defined as the sum of fishing occasions per year (using any gear) from respondents who reported having caught perch (505 000 ± 124 000 ‘gear days’) and pikeperch, (83 000 ± 64 000) respectively.

## Results

**Fig. 1:**
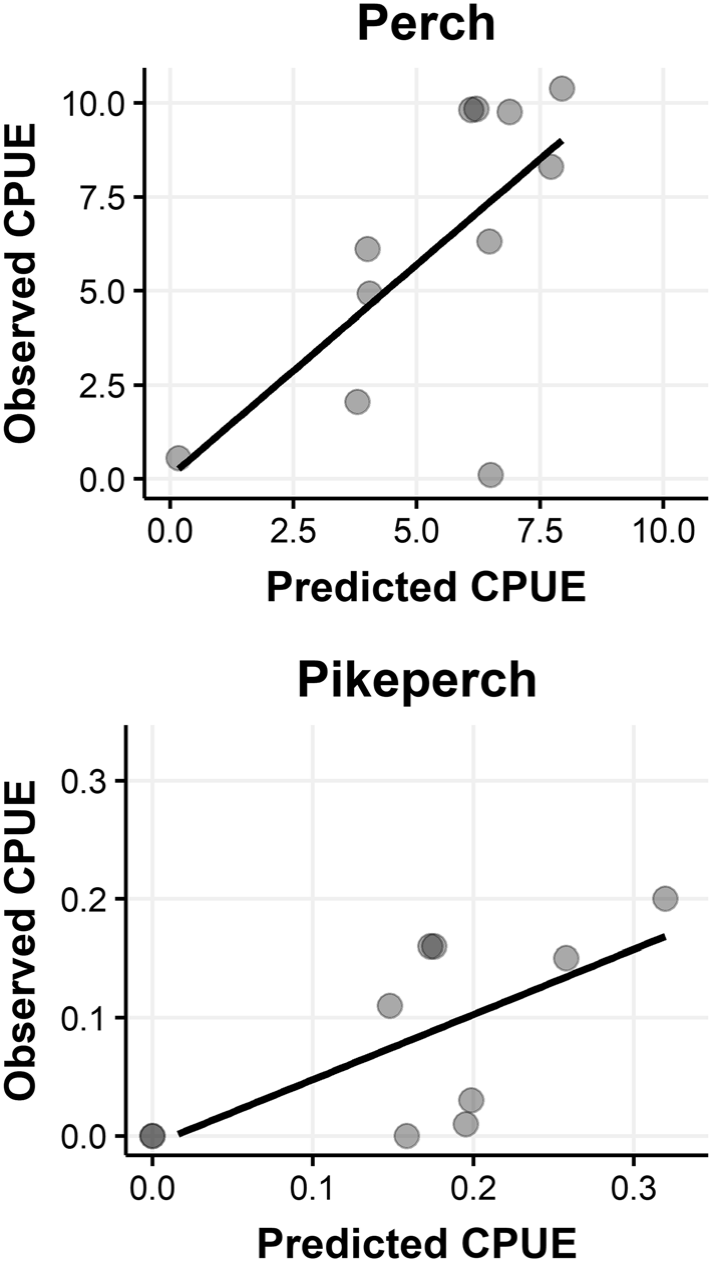
Regression relationships for spatial predictions of CPUE (number per net and night) and observed CPUE in standardized monitoring for perch and pikeperch. Note that points (monitoring areas) can overlap, as indicated by darker grey)

The CPUE of large perch and pikeperch predicted by the habitat productivity functions had a strong fit to the CPUE observed at monitoring sites (Fig. 1). Average predicted CPUE was 5.4 (SD = 2.3) for perch and 0.16 (SD = 0.10) for pikeperch, while observed CPUE was 6.2 for perch (SD = 3.9) and 0.08 for pikeperch (SD = 0.08). Linear regression models (*y* = *ax* + *b*, where *y* = observed CPUE and *x* = predicted CPUE) resulted in, for perch: *a* = 1.1 ± 0.4 (SE), *b* = 0.06 ± 2.5 (SE; *n* = 11) and explained 44% of the variation in observed CPUE (*p* = 0.027, *F*_(1, 9)_ = 7.0), and for pikeperch: *a* = 0.54 ± 0.21 (SE), *b* = − 0.007 ± 0.04 (SE, *n* = 10) and explained 45% of the variation in observed CPUE (*p* = 0.035, *F*_(1, 8)_ = 6.4).

**Fig. 2:**
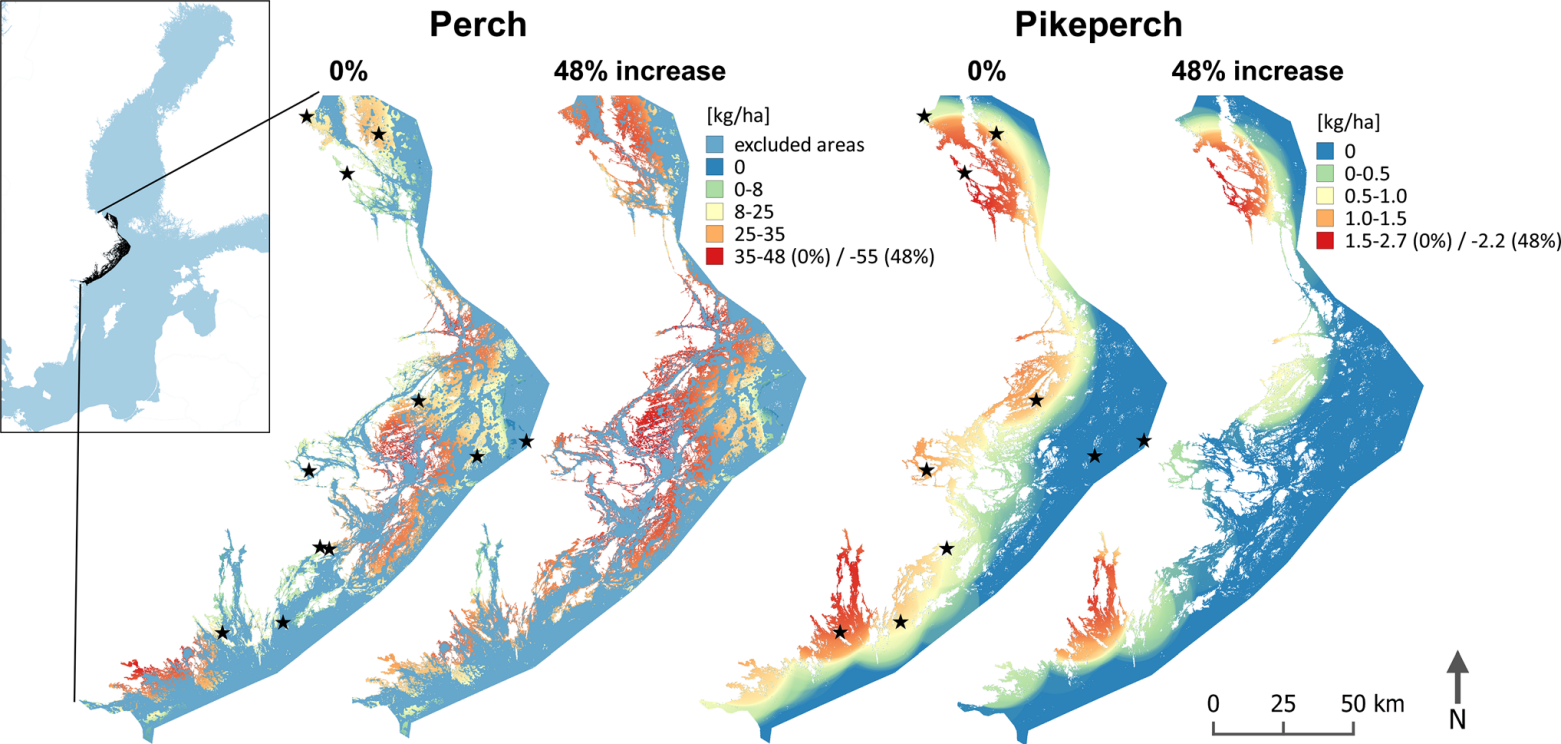
Estimated biomass distribution (kg ha^−1^) of perch > 20 cm (left) and pikeperch > 30 cm (right). Biomass estimates were based on spatial predictions of recruitment habitats (Bergström et al. 2013) combined with habitat productivity functions (Sundblad et al. 2014) under a set of eutrophication scenarios. The maps show average scenarios from three different modelling techniques under initial Secchi depth conditions (0% change) and a 48% increase, which corresponds to the reference level for water clarity in the Baltic Sea Action Plan. Stars denote the location of standardized gillnet monitoring areas used for validation of the biomass predictions (Fig. 1). Note that the maximum biomass per hectare for each species differs between scenarios (legends), and that areas deeper than 10 m depth have been excluded for perch. The inset shows the location of the study area within the Baltic Sea

The resulting fish biomass maps show that pikeperch is mainly concentrated to large bays close to the mainland, corresponding to areas typically characterized by higher eutrophication levels (lower Secchi depth). Perch biomass was more evenly distributed across the archipelago, however with a dominance in the middle parts (Fig. 2). Calculated for the entire study area, average predicted biomass of perch > 20 cm under the initial scenario (0% change) was 9.4 kg per hectare (± 0.33 SD) and of pikeperch > 30 cm it was 0.65 kg per hectare (± 0.48 SD, Fig. 2).

**Fig. 3:**
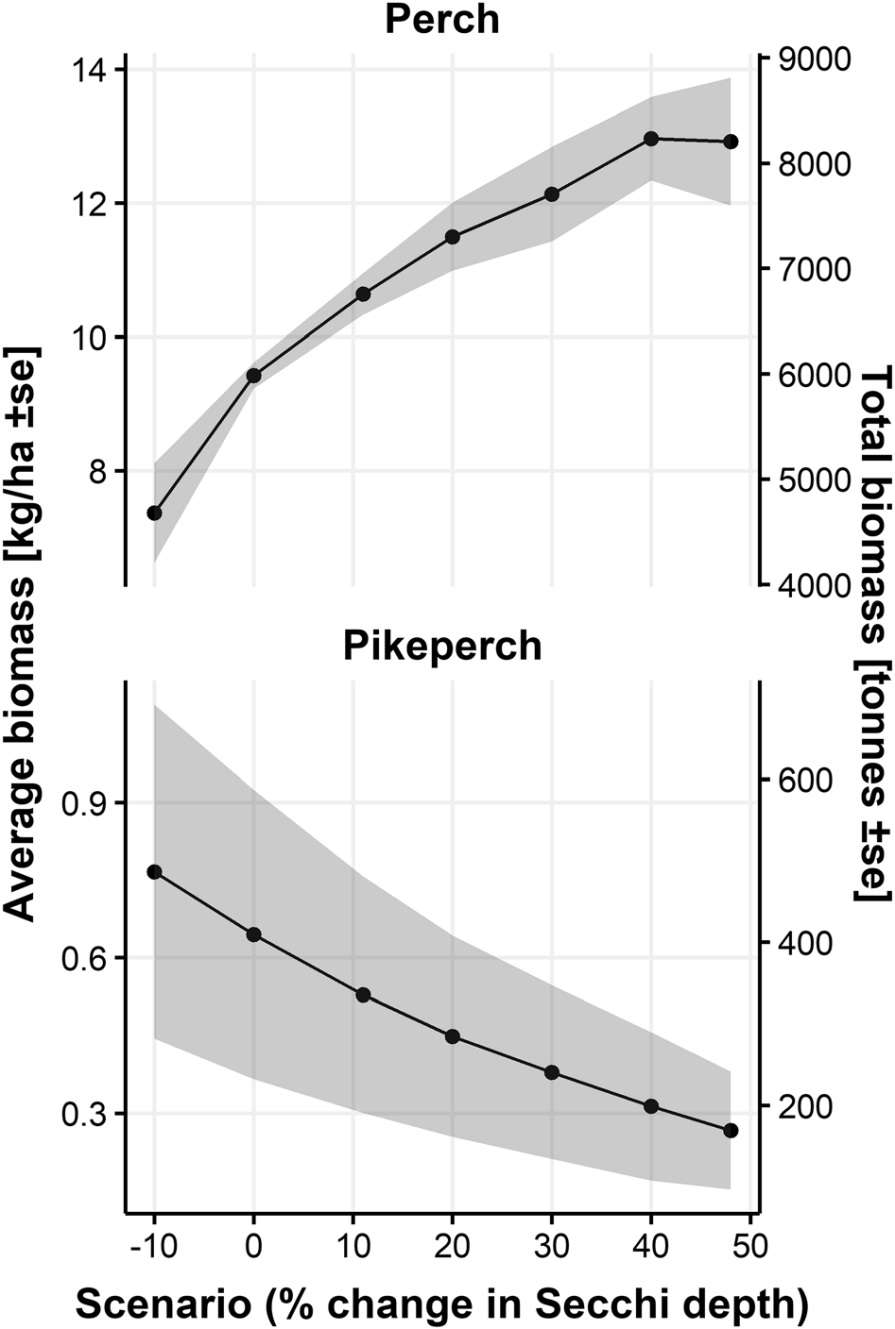
Average (per hectare; left axis) and total (for whole study area; right axis) predicted biomass of perch (> 20 cm) and pikeperch (> 30 cm) at different eutrophication levels within the studied archipelago area. Uncertainty (grey areas, 1 standard error) was calculated across three different modelling techniques (see text)

An increase in Secchi depth, indicating a reduction in the level of eutrophication, was predicted to increase the biomass of perch and reduce the biomass of pikeperch (Fig. 3).

**Fig. 4:**
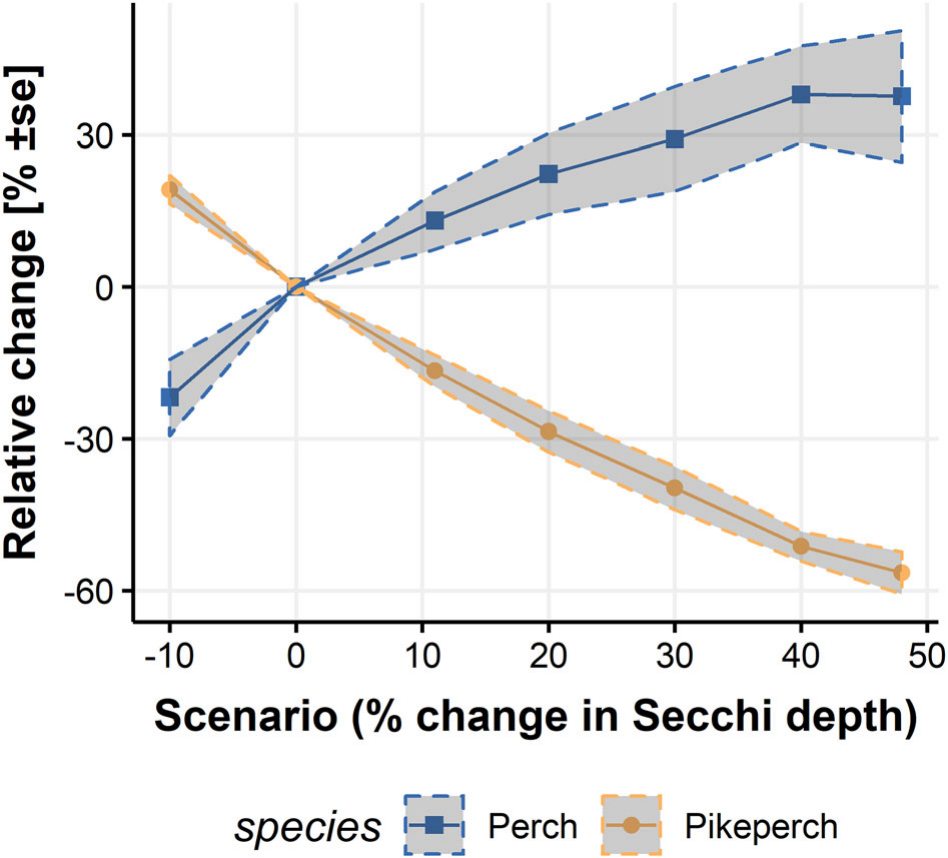
Relative change (%) in coastal predatory fish biomass within the studied coastal area, at different eutrophication levels. Uncertainty (grey areas, 1 standard error) was calculated across three different modelling techniques (see text)

The results from the TCM study (Söderqvist et al. 2005) indicated a mean WPUE in recreational fishing of 0.8 kg per fishing hour for perch (SD 1.5) and 0.3 kg per fishing hour for pikeperch (SD 0.6) during the spring of 2002. For both species there was a statistically significant positive relationship between WPUE on the probability that a particular fishing site was selected (Söderqvist et al. 2005). The mean WTP for one additional kg of fish per fishing hour was estimated in 2002 prices at SEK 71.6 for perch and SEK 153 for pikeperch, which corresponds to EUR 8.29 and EUR 18.0, respectively, in 2018 prices after adjusting for inflation and applying an exchange rate of EUR 1 = SEK 10.2567. Hence, pikeperch showed to be more highly valued than perch. However, as the WPUE for pikeperch was lower, a 1 kg increase corresponds to a substantially higher relative increase (1.3/0.3 = 333%) compared to for perch (1.8/0.8 = 125%). In comparison to these estimates, more moderate changes are suggested by the scenario analyses (Fig. 4). For example, an improvement of water clarity to the level of the BSAP target (11% increase) was suggested to lead to a 13% increase of perch biomass (Fig. 4). Assuming linearity between the predicted biomass and WPUE in the recreational fisheries, as well as between WPUE and WTP, this would correspond to an increase of 0.8 * 13% = 0.10 kg perch per fishing hour, and the WTP for this increase corresponds to 0.10 * 8.29 = EUR 0.8 for perch. Following the same assumptions the WPUE of pikeperch would decrease with 18% as a result of increased water clarity (Fig. 4), giving a decrease of 0.3 * 18% = 0.05 kg per fishing hour. This corresponds to an economic loss of 0.05 * 18.0 = EUR 0.9. The results show that for the recreational value of fishing of these species, the increase in water transparency entails an economic gain in perch that is countervailed by a loss in pikeperch. However, as perch is 6.1 (± 5.0, 95% confidence interval) times more often targeted by recreational fishers (SwAM 2019), a net economic benefit of eutrophication mitigation can be expected with respect to these two fish species.

## Discussion

Our analyses outline how an improved water clarity, in accordance with political commitment to improve the eutrophication status of the Baltic Sea, can affect the distribution of coastal fish recruitment habitats, and how this may change the prevalence of large predatory fish and the extent of recreational fisheries in the coastal zone. By combining species distribution modelling with habitat productivity functions, scenarios for changes in the focal fish populations were mapped, enabling a spatially explicit evaluation. By extending the assessment to impacts on the economic value for the recreational fisheries sector, we illustrate how ecological scenarios for environmental management can be integrated with analyses of economic impacts in a quantitative analysis.

The analyses serve a dual purpose, considering ecological as well as economic aspects of eutrophication mitigation, focusing on potential impacts on the abundance of large predatory fish and on recreational fisheries, respectively. In addition to the effects on economic value associated with recreational fisheries, effects on other types of ecosystem services can also be anticipated. Large predatory fish represent a key ecosystem component in the coastal zone, with a strong contribution to ecosystem function as well as to provisioning and regulating ecosystem services (Holmlund and Hammer 1999; de Groot et al. 2002). In their environment, large predatory fish regulate the presence of other species via predation. Prey species typically regulated by large predatory fish are medium sized mesopredatory fish, which are observed to expand in population sizes under conditions of reduced predator availability (Ritchie and Johnson 2009; Bergström et al. 2019). One recent example from the study area shows a connection between decreasing predator abundance and dominance of sticklebacks in coastal areas, causing unexpected and detrimental effects on the food web (Donadi et al. 2017). As a result of trophic cascades, the regulation of prey by predatory fish species indirectly affects primary producers, counteracting excessive occurrences of ephemeral filamentous algae (Eriksson et al. 2009; Donadi et al. 2017). Thereby, the predatory fish may contribute to relieving eutrophication symptoms. Meta-analyses indicate that changes in this regulation from large predatory fish can be as important as changes in nutrient loadings for controlling the presence of nuisance algae (Östman et al. 2016). In relation to human well-being, predatory fish also contribute through the provisioning of food and cultural values enabled by commercial and recreational fisheries. The current study focused on the recreational fishing sector, which is dominating over commercial fishing in the studied coastal areas (SwAM 2019).

Although ecological feedback loops can be expected to increase the quantitative uncertainty of the presented predictions, the overall conclusions of our study, showing increasing perch and decreasing pikeperch under reduced eutrophication, appear likely also in relation to a mechanistic understanding of the fish species physiological responses to water clarity (Sandström and Karås 2002; Ljunggren and Sandström 2007; Veneranta et al. 2011), as well as with changes observed in fish populations in the Baltic Sea during the decades when eutrophication increased (Bergström et al. 2013). As such, results from scenario analyses are useful for providing examples of likely management outcomes. Here, the predictive models, based on empirically assessed quantitative relationships, provide a basis for economic analyses in support of an integrated strategic evaluation.

A key factor enabling this study was the combination of a number of quantitative analyses based on unique empirical data covering various temporal and spatial scales as well as different disciplines. However, such an extended scope may also mean that more sources of uncertainty are introduced. For instance, the value of the recreational fisheries stems from surveys performed in the Stockholm archipelago in 2002 (Söderqvist et al. 2005), while the national fishing habits and species preferences originates from surveys performed 2013–2017, on a larger scale (the Baltic Proper, SwAM 2019). Another important potential source of uncertainty is the fish biomass map predictions. These were evaluated by (i) comparing predicted CPUE with results for specific monitoring sites across the study area and (ii) by comparing the resulting biomass estimates for the entire study area with independent biomass assessments. The evaluation of predictions against monitoring data showed an overall good predictive ability, although the predicted CPUE of pikeperch was biased towards higher than observed values (Fig. 1). This overestimation influences the absolute values obtained (Figs. 2, 3), but are expected to have smaller impact on the relative changes in biomass (Fig. 4) and on the economic assessment, which rely on relative changes and WTP for change in WPUE in the recreational fisheries (Söderqvist et al. 2005). Regarding the biomass estimates, few studies were available for comparison. In two well-studied coastal sites in the northern part of the study area (Forsmark and Kallrigafjärden), perch biomass has previously been estimated at 34 and 30 kg ha^−1^ and pikeperch biomass at 3.7 and 6.6 kg ha^−1^ (Heibo and Karås 2005), for fish above approximately 10 cm. In the central Baltic, south of the study area, biomass of perch > 10 cm in the summer has been estimated at 38 kg ha^−1^ in an enclosed coastal site (Adill and Andersson 2006). Including also smaller fish, the same biomass density has been found in a comparison of 100 fish populations in 38 lakes (Downing and Plante 1993). A direct comparison is difficult as the cited estimates are related to particular sites primarily consisting of suitable habitats, while our study encompasses also large parts of the outer archipelago, where recruitment habitats are scarce and predicted biomasses were very low (Fig. 2), thus lowering averages for the entire study area over which the population is distributed. Additionally, different length classes of fish have been included. In order to provide more direct comparisons, we utilized length frequency distributions from monitoring in the study area and calculated the proportion of fish 10–20 and > 20 cm for perch, and 10–30 and > 30 cm for pikeperch. Based on these proportions the average (across the entire study area) predicted biomass of fish > 10 cm was 16 kg ha^−1^ for perch and 1.5 kg ha^−1^ of pikeperch. Additionally, a direct comparison was possible for a subset of the study area, which overlapped with one of the previously published results (Forsmark and Kallrigafjärden). In this subset, length correction of predicted biomasses yielded for perch 50 and 45 kg ha^−1^ for each subarea respectively, and for pikeperch 1.2 and 4.2 kg ha^−1^ respectively, which is more similar to Heibo and Karås (2005).

The evaluation of management scenarios identifies potential trade-offs when several ecosystem and economic aspects are included. Here, a gain of the perch recreational fisheries was predicted to be countervailed by a loss in the pikeperch fisheries, when WTP for a changed WPUE was considered individually for the two species. However, when scaling the results to the volume of recreational fishing on both species, a total net gain was predicted, since the total number of fishing days targeting perch is much higher than for pikeperch. The results imply an economic benefit from mitigating eutrophication from a recreational fisheries perspective. A full economic valuation should also consider other species of relevance, as well as impacts on other ecosystem services, and take into account the fact that use values such as recreational values are only one type of economic value associated with a particular ecosystem service; non-use values such as existence values should typically be added (Freeman et al. 2014). As one example, Northern pike (*Esox lucius*) is highly targeted by the recreational fisheries, but could not be included here since habitat productivity functions are lacking for this species. Still, knowledge of the ecology of pike suggests that a similar response to eutrophication mitigation as for perch could be expected (Sandström et al. 2005; Engström-Öst and Mattila 2008; Salonen et al. 2009), implying that the net benefit for recreational fisheries could be larger than the estimates presented. Including additional ecosystem services associated to predatory fish, such as biological regulation (Donadi et al. 2017), would likely also contribute to increase the estimated benefits of eutrophication mitigation, supporting previous results which show net benefits as the most common overall outcome (Czajkowski et al. 2015; Scharin et al. 2016; Bauer et al. 2018).

In conclusion, our analyses demonstrate the usefulness of integrating economic information with quantitative ecological predictions in a spatial context. Combining the spatial approach with scenario analyses is beneficial as it allows mapping potential trade-offs and net outcomes of management alternatives at both more detailed and large-scale levels.
